# Hydrogen Sulfide Overproduction Is Involved in Acute Ischemic Cerebral Injury Under Hyperhomocysteinemia

**DOI:** 10.3389/fnins.2020.582851

**Published:** 2020-11-30

**Authors:** Yan Ji, Yusheng Li, Zichen Zhao, Panxing Li, Yi Xie

**Affiliations:** Department of Neurology, The First Affiliated Hospital of Zhengzhou University, Zhengzhou, China

**Keywords:** ischemia, homocysteine, hydrogen sulfide, inflammatory response, mitochondria

## Abstract

**Objectives:**

This study aimed to identify the involvement of hydrogen sulfide overproduction in acute brain injury under ischemia/reperfusion and hyperhomocysteinemia.

**Methods:**

*In vitro* and *in vivo* experiments were conducted to determine: the effect of sodium hydrosulfide treatment on the human neuroblastoma cell line (SH-SY5Y) under conditions of oxygen and glucose deprivation; the changes of hydrogen sulfide levels, inflammatory factors, energetic metabolism, and mitochondrial function in the brain tissue of rats under either ischemia/reperfusion alone or a combination of ischemia/reperfusion and hyperhomocysteinemia; and the potential mechanism underlying the relationship between homocysteine and these changes through the addition of the related inhibitors. Furthermore, experimental technologies, including western blot, enzyme-linked immunosorbent assay, immunofluorescence, reverse transcription polymerase chain reaction, and flow cytometry, were used.

**Results:**

Our study found that high concentration of sodium hydrosulfide treatment aggravated the decrease in mitochondrial membrane potential, the increase in both mitochondrial permeability transition pore and translocation of cytochrome C, as well as the accumulation of reactive oxygen species in oxygen and glucose deprived SH-SY5Y cells. As a result, neurological deficit appeared in rats with ischemia/reperfusion or ischemia/reperfusion and hyperhomocysteinemia, and a higher water content and larger infarction size of cerebral tissue appeared in rats combined ischemia/reperfusion and hyperhomocysteinemia. Furthermore, alterations in hydrogen sulfide production, inflammatory factors, and mitochondria morphology and function were more evident under the combined ischemia/reperfusion and hyperhomocysteinemia. These changes were, however, alleviated by the addition of inhibitors for CBS, CSE, Hcy, H_2_S, and NF-κB, although at different levels. Finally, we observed a negative relationship between the blockage of: (a) the nuclear factor kappa-B pathway and the levels of cystathionine β-synthase and hydrogen sulfide; and (b) the hydrogen sulfide pathway and the levels of inflammatory factors.

**Conclusion:**

Hydrogen sulfide overproduction and reactive inflammatory response are involved in ischemic cerebral injury under hyperhomocysteinemia. Future studies in this direction are warranted to provide a scientific base for targeted medicine development.

## Introduction

Hydrogen sulfide (H_2_S) is an established toxic gas that is characterized by an unpleasant odor of rotten eggs (Wang, 2012). Furthermore, physiological and pathological effects of H_2_S as an endogenous signaling molecule have also been described (Fu et al., 2012; Zhang et al., 2015). Indeed, H_2_S is identified as the third gaseous transmitter after nitric oxide (NO) and carbon monoxide (CO) (Wang, 2002). Endogenous H_2_S is mainly synthesized from both cysteine (Cys) and homocysteine (Hcy), which are catalyzed by the cystathionine β-synthase (CBS) and cystathionine γ-lyase (CSE) enzymes. In fact, Hcy (i.e., an endogenous sulfur-containing amino acid and a metabolite of the essential amino acid methionine), is metabolized by two pathways, namely the methylation and transsulfuration pathways, and the latter is linked to endogenous H_2_S formation.

H_2_S plays several roles in mammals as a gaseous signal molecule. For example, the activity of the *N*-methyl-D-aspartate (NMDA) receptor, the induction of hippocampal long-term potentiation, the relaxation of vascular smooth muscles and the regulation of blood pressure have been reported to be associated with the physiological concentration of H_2_S (Yang et al., 2008; Wang et al., 2015; Tu et al., 2016). Actually, the concentration of H_2_S in the mammalian brain is challenging to measure and was reported to vary greatly in previous studies according to the assessment methods utilized (Ishigami et al., 2009; Levitt et al., 2011). In addition, a direct metabolic coupling between H_2_S and oxygen (O2) has been described, i.e., while H_2_S production is independent from O_2_, its metabolism is O_2_-dependent. Specifically, although the intracellular H_2_S concentration is maintained at low levels through concomitant oxidation, it increases when its oxidation is outbalanced by its production during hypoxia. In fact, permanent occlusion of the middle cerebral artery (MCAO) was shown to increase the endogenous H_2_S level in the ipsilateral cortex. This was especially valid for rat that received simultaneous intraperitoneal injection of Cys (Qu et al., 2006). Accumulated evidence also suggests a possible association between the concentration and the effects of H_2_S. Specifically, high levels of H_2_S are known to cause the generation of both reactive oxygen species (ROS) and reactive nitrogen species (RNS), whereas lower amounts react with hydrogen peroxides (H_2_O_2_), peroxynitrite (ONOO^–^), and oxide ion (O^2–^) (Mikami et al., 2011). In addition, some studies found H_2_S to be involved in inflammatory responses (Bhatia, 2012). Specifically, biphasic effects of H_2_S on inflammatory signals in murine lipopolysaccharide (LPS)-treated macrophages were reported (Whiteman et al., 2010). Therefore, future studies to better define this relationship are required given the contradictory findings in the literature, i.e., while the administration of sodium hydrosulfide (NaHS, an H_2_S donor) significantly increases the infarct volume after MCAO in rats (Ishigami et al., 2009), beneficial effects of H_2_S (e.g., decrease in I/R injury and localized inflammatory reaction) can also be observed (Huang et al., 2017). Indeed, the facticity and mechanism associated with the various H_2_S effects remain to be defined and future studies should address these questions.

In a previous cohort study, we observed a prominent neurological deficit and poor prognosis in patients with both acute ischemic stroke and elevated levels of plasma Hcy (Ji et al., 2015). Given that these findings urged us to explore the possible pathological mechanism implicated, we performed the present study to observe the effects of different concentration of H_2_S on SH-SY5Y cells under oxygen and glucose deprivation (OGD). Furthermore, the changes in H_2_S levels, inflammatory factors, energetic metabolism, and cerebral mitochondrial function in rats under I/R alone or a combination of I/R and hyperhomocysteinemia (HHcy) were assessed. Finally, the possible pathologic association between H_2_S levels and deteriorated ischemia/reperfusion (I/R) brain injury in patients with HHcy was discussed.

## Materials and Methods

**FIGURE 1:**
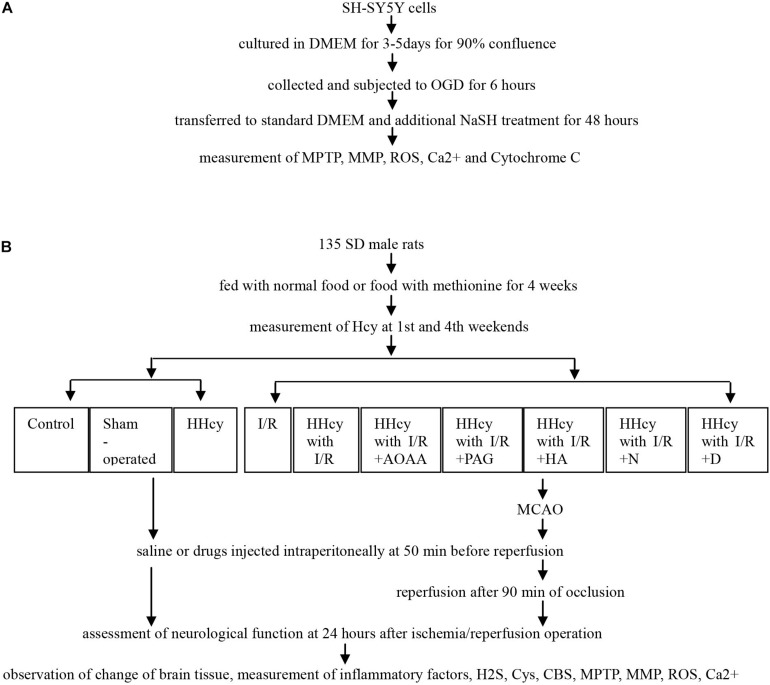
Flowchart of experimental process. **(A)** SY5Y cells were cultured and subjected to OGD and different NaSH treatment, and then collected to measurement of MPTP, MMP, ROS, Ca^2+^ and Cytochorme C. **(B)** SD males fed using normal food or food with methionine were divided into ten groups, and received MCAO or not, saline or inhibitor injected intraperitoneally, and finally assessed for neurological function, morphological change and cytokines levels. *HHcy, hyperhomocysteinemia; I/R, ischemia/reperfusion; AOAA, aminooxyacetic acid; PAG, DL-propargylglycine; HA, hydroxylamine; N, N-anandamide; D, Pyrrolidine dithiocarboxylic acid ammonium salt.

The study was designed as two approaches: the *in vitro* and *in vivo* studies, and the related experimental process was demonstrated in Figure 1.

### Antibodies and Reagents

L-methionine, Cys, DL–propargylglycine (PAG), aminooxyacetic acid (AOAA), NaHS, hydroxylamine (HA), *N*-anandamide, and pyrrolidine dithiocarboxylic acid ammonium salt (PDTC) were obtained from Sigma. In contrast, the anti-rabbit horseradish peroxidase (HRP)-conjugated secondary antibodies were purchased from Santa Cruz, whereas the anti-rabbit β-actin antibody and Cytochrome c oxidase IV (COXIV) were developed by Proteintech. In addition, the anti-mouse nuclear factor kappa-B (NF-κB) p65 and glyceraldehyde-3-phosphate dehydrogenase (GAPDH), anti-rabbit histone H3 antibodies were purchased from Abcam, while the anti-rabbit cytochrome C, IkB-alpha (IκBa), P-IκBa and CBS antibodies were obtained from CST. Furthermore, while the primers were procured from Western Biotechnology, the JC-1 mitochondrial membrane potential (MMP), Fluo-3AM (calcium, Ca^2+^), and ROS assay kits were purchased from BD Biotechnology. The enzyme-linked immunosorbent assay (ELISA) kits for interleukin (IL)-6, -1 and -8 were obtained from Like Beijing, whereas the assay kits for Cys, H_2_S, adenosine triphosphate (ATP), and Hcy were acquired from Jiancheng Nanjing. Finally, while the kits for Nuclear and Cytoplasmic Protein Extraction and mitochondria isolation were purchased from Beyotime, those for the mitochondrial permeability transition pore (MPTP) were obtained from GENMED. All drugs were dissolved in saline and administered to rats through intraperitoneal injection.

### Animals and Experimental Grouping

Total 135 Male Sprague-Dawley rats (230–260 g) from Chongqing Ensville Biotechnology Co., Ltd. were housed individually with the following conditions: natural light/dark (12/12 h) cycles, temperature of 22–24°C, either normal food or food with 1.7% methionine were provided, and water was available *ad libitum* for 4 weeks. All procedures were reviewed and approved by the Institutional Animal Care and Use Committee of the University of Zhengzhou, School of Medicine in accord with Animal Care and Use Program Guidelines of the National Institutes of Health. Rats were randomly assigned to the following groups in Table 1.

**TABLE 1:** The detailed data of experiment groups of SDA rats.

Group	*n*	Food	Operation	Treatment
C (control group)	10	Normal	No	Saline (1 ml/kg)
S (sham-operated group)	10	Normal	Sham-operated	Saline (1 ml/kg)
I/R (ischemia/reperfusion group)	15	Normal	I/R operation	Saline (1 ml/kg)
H (HHcy group)	10	Methionine	No	Saline (1 ml/kg)
HI/R (HHcy with I/R)	15	Methionine	I/R operation	Saline (1 ml/kg)
A (HI/R + AOAA group)	15	Methionine	I/R operation	AOAA (1 ml/kg, 10 mg/1 ml)
P (HI/R + PAG group)	15	Methionine	I/R operation	PAG (1 ml/kg, 45 mg/1 ml)
O (HI/R + HA group)	15	Methionine	I/R operation	HA (1 ml/kg, 12.5 mg/1 ml)
N (HI/R + *N*-anandamide group)	15	Methionine	I/R operation	*N*-anandamide (1 ml/kg, 0.2 mg/1 ml)
D (HI/R + PDTC group)	15	Methionine	I/R operation	PDTC (1 ml/kg, 150 mg/1 ml)

*HHcy, hyperhomocysteinemia; AOAA, aminooxyacetic acid; PAG, DL–propargylglycine; HA, hydroxylamine; PDTC, pyrrolidine dithiocarboxylic acid ammonium salt.*

### Cells Culture and Treatment

SH-SY5Y neuroblastoma cells were purchased from the Shanghai Cell Bank of the Chinese Academy of Sciences. Successively, they were cultured in high glucose Dulbecco’s Modified Eagle Medium (DMEM), supplemented with 10% fetal bovine serum (FBS) and 1% of a penicillin-streptomycin stock, at 37°C in humidified air containing 5% carbon dioxide (CO_2_). The serum- and glucose-free DMEM was bubbled with a mixture of 95% nitrogen (N_2_) and 5% CO_2_ for 30 min and an anaerobic acrylic jar was supplied with the same gas for 6 min. Thereafter, the cells were incubated in the DMEM and then were placed in the jar. The jar was sealed rapidly afterward and placed in an incubator at 37°C. Finally, following 6 h of OGD, the cells were transferred to standard culture conditions and subjected to either a 0, 100, 500, 1,000, or 1,500 μmol/L additional NaSH treatment for 48 h.

### I/R Operation and Neurological Function Assessment

Transient brain ischemia was induced through MCAO, as previously described (Zan et al., 2014). In brief, rats were anesthetized with 7% chloral hydrate (0.5 ml/100 g), while their core temperature was maintained at 36.5–37.5°C through a temperature controller pad during the surgery. Following the separation of the right carotid artery from the surrounding tissue, a 3–0 monofilament nylon suture was advanced from the external into the internal carotid artery, until the origin of the middle cerebral artery was blocked. After 90 min of occlusion, the suture was withdrawn to restore blood flow, while either saline or drugs were injected intraperitoneally at 50 min before reperfusion. The wound was then sutured, and rats were allowed to recover from anesthesia before returning to their cages. While sham-operated rats underwent the same procedure, arterial occlusion was not conducted. Animals that died prematurely were excluded from the study. The Zea Longa scale was used for independently evaluating the neurological deficit of rats at 24 h after the operation by two researchers.

### Measurement of Water Content and Infarct Size of Brain Tissue

Rats were decapitated and the whole brain tissue was obtained 24 h after operation. Thereafter, the cerebral tissue was isolated from the olfactory bulb, cerebellum and brainstem, and weighed by an electronic analytical balance (i.e., wet weight). Successively, brain tissues were dried at 65°C for 36–72 h to a constant quality (i.e., the difference between two weighings is less than 1%) in an oven and weighed again (i.e., dry weight). Finally, the brain water content was calculated as the ratio of wet over dry weight.

Similarly, after obtaining the cerebral tissue, coronal sections (2 mm) were prepared. Briefly, they were stored for 5 min in a freezer at −80°C and were then stained with a 1% 2,3,5-triphenyltetrazolium chloride (TTC) solution at 37°C for 15 min. Thereafter, they were fixed using 4% formaldehyde for 24 h and photographed with a digital camera. While the infarct size was traced and quantified with the ImageJ software (National Institutes of Health, Bethesda, MD, United States), it was expressed as a percentage of the bilateral hemisphere.

### Transmission Electron Microscope (TEM) Observation for Mitochondrial Morphology

Cerebral tissues were fixed using 2.5% glutaraldehyde for 24 h and then sliced into fragments of 1 mm^3^ in size. Subsequently, they were fixed, dehydrated, embedded, cut into 1 μm-thick slices and further dried. Thereafter, they were sliced into ultraslices of 50 nm and finally stained with both uranyl acetate and lead citra to observe the changes in mitochondrial morphology using a transmission electron microscope (TEM) (PHILIPS Tecani-10).

### Western Blot for Cytochrome C, IκBa, p-IκBa, NF-κB, and CBS Protein Levels

SH-SY5Y cells were cultured as abovementioned, and then were collected and washed in an ice-cold phosphate buffer saline (PBS). Cell mitochondria were isolated according to the manufacturer’s protocol on the mitochondria isolation Kit for measuring cytochrome C protein level in both the mitochondria and cytoplasm separately.

Brain tissues were cut into tiny fragments and put into the homogenate tube, together with 1 ml of RIPA per 100 mg of tissue. The tissues were then homogenized for 20 times and transferred into a centrifuge tube (1.5 ml). Thereafter, the supernatant was collected after a 10 min-long centrifugation (12,000 rpm) at 4°C. To measure the NF-κB protein level, nuclear and cytoplasmic proteins were separately extracted, according to the manufacturer’s protocol on the Nuclear and Cytoplasmic Protein Extraction Kit.

Protein concentration was measured using the Bradford assay. Briefly, equal amounts of protein (30 μg) per lane were separated through a 12% sodium dodecyl sulfate–polyacrylamide gel electrophoresis and then transferred onto polyvinylidene difluoride membranes. Thereafter, membranes were incubated with the rabbit anti-cytochrome C (1:2000), rabbit anti-IκBa (1:500), rabbit anti-p-IκBa (1:500), rabbit anti- NF-κB (1:500), rabbit anti-CBS (1:500), goat anti-GAPDH (1:1000), mouse anti-histone H3 (1:1000), rabbit anti-COXIV (1:1000) and rabbit anti-β-actin (1:2000) antibodies at 4°C overnight. This was followed by incubation with secondary antibodies for 2 h at room temperature. Finally, blots were scanned with a light/ultraviolet gel scanning system (Tannon-4200, China) and quantified with an analytic soft system (Labworks^TM^ Analysis Software, United States). Protein levels were normalized to GAPDH, β-actin, COXIV and histone H3.

### Measurement of Hcy, IL-1,-6, -8, H_2_S, Cys, and ATP Levels in Brain Tissue

Both a plasma sample from the angular vein and a cerebrospinal fluid sample were obtained from rats that received either normal food or food with methionine after 12 h of fasting at the 1st and the 4th weekends. While a capillary tube was rotated into rats’ angular vein to obtain the blood samples, cerebrospinal fluid samples were drained from the foramen magnum (exposed through an operation) after rats were anesthetized. The Hcy levels were measured using Hcy assay kits, according to the manufacturer’s instructions.

Brain tissue samples were kept in liquid nitrogen, thawed and then added to PBS in a 1:9 ratio. Thereafter, samples were homogenized and centrifuged at 2,000 rpm for 20 min. The supernatant was then carefully collected and the IL-1, -6, and -8 levels were determined using ELISA analysis kits. Tissues were homogenized as mentioned above. To measure H_2_S levels, samples were centrifuged (12,000 rpm) at 4°C for 10 min and the supernatant was collected. Thereafter, levels of H_2_S were determined using the H_2_S assay kit. In contrast, samples were centrifuged (8,000 rpm) at 4°C for 10 min to assess Cys levels. Subsequently, the supernatant was collected and Cys levels were determined using the Cys Assay Kit. Finally, ATP levels were measured. Samples were boiled in water for 10 min, they were then mixed and extracted for 1 min. This was followed by centrifugation at 3,500 rpm for 10 min and collection of the supernatant. ATP levels were assessed using the ATP Assay Kit (Colorimetric). All procedures followed the manufacturer’s instructions on the reagent kits.

### Real-Time Polymerase Chain Reaction (RT-PCR) for CBS mRNA

Brain tissues were cut into tiny fragments and mixed with 1 ml of TRIzol reagent (Beyotime) per 100 mg of tissue. They were then homogenized, dissolved and centrifuged (12,000 rpm) at 4°C for 10 min. Thereafter, the supernatant was collected and added to 0.2 ml of chloroform (Shanghai Chemical Reagent Company). Subsequently, the suspension was centrifuged at 10,000 rpm at 4°C for 10 min and the supernatant was then discarded, and the RNA pellet was washed in 75% ethanol and re-suspended in RNAase-free water. The RNA quantity and integrity were verified by agarose gel electrophoresis. In addition, RNA to cDNA reverse transcription was performed on 1 μg of total RNAs using the iScript Reverse Transcription reagent (Western Biotechnology). Successively, quantitative PCR was performed using the Funglyn Biotech Real Time PCR System (Funglyn Biotech, Canada). To determine the cycle threshold (CT) values for CBS and β-actin, SYBR green PCR reagents (Western Biotechnology) were used. Thereafter, a 35× amplification was performed for 4 min, 20 and 30 s at 94, 94, and 72°C, respectively. β-actin was used as an internal control to normalize mRNA abundance. The sequence of oligonucleotides used for RT-PCR was the following: (a) CBS, sense: CCCACCAACGCCAGATTC and antisense: TGCGGTGTCATCGTAGTGC; and (b) β-actin, sense: CCCATCTATGAGGGTTACGC and antisense: TTTAATGTCACGCACGATTTC. During PCR amplification, the amplified products were continuously measured through the determination of their fluorescence emission. All the gene expression data relative to the PCRs were analyzed using the 2^–ΔΔCt^ method and normalized to the β-actin RNA.

### Assessment of MPTP, ROS, Ca^2+^ and MMP Levels

SH-SY5Y cells were cultured and treated as mentioned above, washed with cleaning solution, then added 500 μl of dying working solution and incubated for 20 min at 37°C according to the protocol on the MPTP assay kit. Finally, cells were observed using an inverted fluorescence microscope (BM, China).

Rats were decapitated and whole brain tissues were obtained 24 h after I/R operation. Following a PBS wash, a blot of cerebral tissue (100 mg) was cut into tiny fragments and mitochondria were obtained using the Tissue Mitochondria Isolation Kit, according to the manufacturer’s protocol. Thereafter, mitochondria were washed with PBS (twice) and centrifuged at 1,500 rpm at 4°C for 5 min. After collecting the supernatant, 200 μl of dying working solution was added and mitochondria were incubated for 30 min at 37°C. Subsequently, they were again washed with PBS (twice) and re-suspended for measuring the mitochondrial permeability transition pore (MPTP) by flow cytometry (CytoFlex, United States).

SH-SY5Y cells and brain tissues were prepared as mentioned above. The cell suspension solution was poured into a centrifuge tube (1.5 ml) and centrifuged (1,500 rpm) at 4°C for 5 min. After collecting the supernatant, the solution was re-suspended in PBS and incubated with a fluorescent probe of DCFH-DA, according to the manufacturer’s protocol on the ROS Assay Kit. Finally, the solution was measured using flow cytometry (BD, United States) to determine the ROS levels.

Similarly, the cell suspension solution were incubated with a fluorescent probe, Fluo-3, according to the manufacturer’s protocol on the Fluo-3 AM kit, and measured using flow cytometry (BD, United States) to determine the Ca^2+^ levels.

Furthermore, cell suspension was prepared as previously mentioned, then incubated with the JC-1 working solution, washed with the JC-1 staining buffer and resuspended in the JC-1 staining buffer, according to the manufacturer’s protocol on the JC-1 MMP Assay Kit. Finally, cells suspension was measured using flow cytometry (BD, United States) to determine the MMP levels.

### Statistical Analysis

All experiments were independently performed in triplicates. Data were summarized by means and standard errors (standard error of the mean; SEM). A one-way ANOVA test, followed by Bonferroni *post hoc* testing, was used to compare groups of three or more. Independent *t*-tests were used for two-group comparisons. *P*-values less than 0.05 were considered statistically significant. All statistical analyses were conducted using SPSS 17.0 (SPSS Inc.).

## Results

### Changes in SH-SY5Y Cells Under the OGD and NaSH Treatment

*In vitro* part, SH-SY5Y cells simultaneously were subjected to OGD and different concentrations of NaSH treatment. Next, they were collected for assessment of the related protein level, changes in mitochondria and energy metabolism.

**FIGURE 2:**
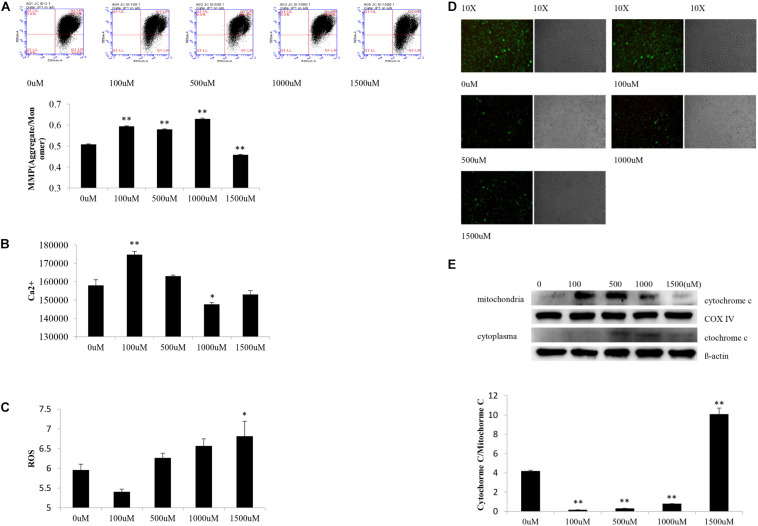
Changes of SY5Y cells subjected to OGD and different concentration NaSH treatment. **(A)** Compared to OGD group, increased MMP levels appeared in cells subjected to OGD and 100, 500, and 1,000 μM NaSH treatment, and decreased MMP levels appeared in cells subjected to 1,500 μM treatment (*n* = 3, *P* < 0.01). **(B)** Compared to OGD group, increased Ca^2+^ levels appeared in OGD and 100 μM of NaSH treatment group (*P* < 0.01) while decreased Ca^2+^ levels appeared in OGD and 1,000 μM of NaSH treated ones (*n* = 3, *P* < 0.05). **(C)** Increased ROS levels appeared in cells subjected to OGD and 1,500 μM treatment compared to OGD group (*n* = 3, *P* < 0.05). **(D)** Compared to OGD group, immunofluorescence showed similar green fluorescence in cells subjected to OGD and 100 μM NaSH treatment, while lower green fluorescence in cells subjected to OGD and 500, 1,000, and 1,500 μM NaSH treatment. **(E)** Compared to OGD group, increased cytochrome C release appeared in cells subjected to OGD and 1,500 μM treatment but decreased in cells subjected to OGD and 100, 500, and 1,000 μM treatment as well (*n* = 3, *P* < 0.01). ^∗∗^*P* < 0.01, ^∗^*P* < 0.05, analyzed by one-way ANOVA and Bonferroni *post hoc* test.

Statistical analyses showed significant difference of MMP levels in SH-SY5Y cells among experimental groups [*F*(4,10) = 581.68, *P* < 0.000]. *Post hoc* testing showed obviously decreased MMP levels in cells subjected to OGD and 1,500 μM of NaSH as opposed to cells treated with OGD alone (*P* < 0.01) (Figure 2A).

Significant difference of Ca^2+^ levels existed among experimental groups [*F*(4,10) = 30.370, *P* < 0.000]. Compare to cells subjected to OGD alone, increased Ca^2+^ levels appeared in OGD and 100 μM of NaSH treated cells (*P* < 0.01) while decreased Ca^2+^ levels appeared in OGD and 1,000 μM of NaSH treated ones (*P* < 0.05) (Figure 2B).

There were significant difference of ROS levels among experimental groups [*F*(4,10) = 6.841, *P* < 0.01]. As opposed to cells subjected to OGD alone, ROS levels increased in cells treated with both OGD and 1,500 μM of NaSH (*P* < 0.05) (Figure 2C).

Different appearance of green fluorescence existed among experimental groups. Compared to cells subjected to OGD alone, cells treated with OGD and 500, 1,000, or 1,500 μM of NaSH showed lower green fluorescence (higher MPTP level) while cells treated with OGD and 100 μM of NaSH showed similar green fluorescence (similar MPTP level) (Figure 2D).

There were significant difference of cytochrome C release in SH-SY5Y cells among experimental groups [*F*(4,10) = 9.238, *P* < 0.01]. Compared to cells treated with OGD alone, increased translocation of cytochrome C existed in cells subjected to OGD and 1,500 μM of NaSH (*P <* 0.01) but decreased translocation of cytochrome C in OGD and 100, 5,00, and 1,000 μM of NaSH treated cells (*P* < 0.01) (Figure 2E).

### Neurological Function Change of Experimental Rats

Total 10 groups of rats were included in the *in vivo* part. Specifically, 105 rats were divided into 7 groups and all received I/R operation. Of these, 8 were excluded from our analysis due to a failure of I/R operation. Additional 10 rats were recruited for each of the other 3 groups. Only one group (i.e., 10 rats) received the sham-operation, and all rats survived. As a result, a total of 127 rats were considered for analysis.

**FIGURE 3:**
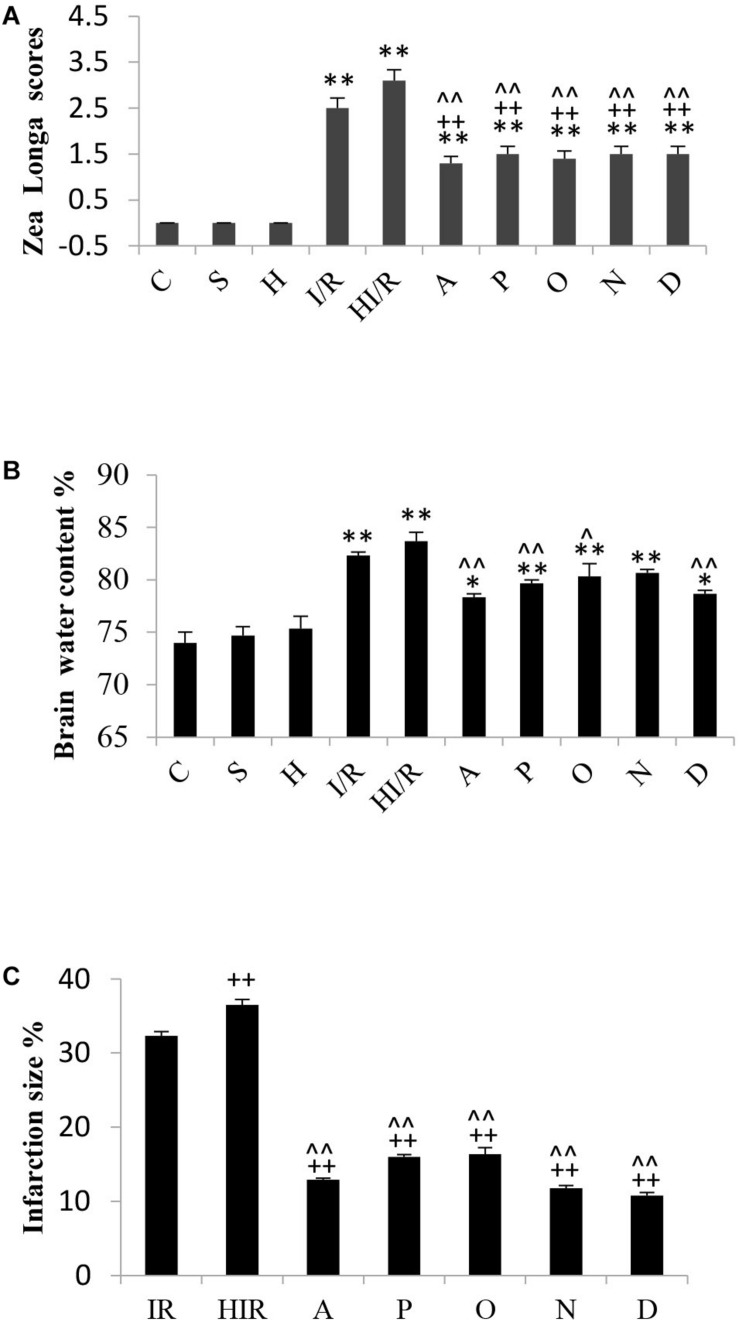
Changes of brain tissue and neurological function in rats 24 h after I/R or not. **(A)** A significant increase of Zea Longa scores appeared in I/R and HI/R groups compared to control groups, especially in the latter, and decreased by all inhibitors (*n* = 10, *P* < 0.01). **(B)** The water content of brain tissue increased evidently in I/R and HI/R groups compared to control groups (*P* < 0.01), and decreased in A, P, O, and D groups when compared to HI/R group (*n* = 3, *P* < 0.05 or 0.01). **(C)** The infarction size of cerebrum tissue in HI/R or I/R group was the first and second largest, separately, and decreased by all inhibitors (*n* = 3, *P* < 0.01). ^∗∗^ or ^∗^ means *P* < 0.01 or 0.05 versus control, ^++^or ^+^ means *P* < 0.01 or 0.05 versus I/R group, and ^∧∧^ or ^∧^ means *P* < 0.01 or 0.05 versus HI/R group, analyzed by one-way ANOVA and Bonferroni *post hoc* test.

The assessment of rats’ neurological function according to Zea Longa scores was performed by 2 independent investigators 24 h after I/R operation. Zea Longa scores were identified under 5-scores grades. The detail was as following: zero point means no neurological impairment, one point means a failure of complete stretch of leg, two points means leg bucking, three points means turning to one side slightly in walking, four points means turning to one side severely in walking, five points means falling to one side in walking. Statistical analyses showed significant difference of Zea Longa scores among experimental groups [*F*(9,90) = 46.150, *P* < 0.000], and *post hoc* testing showed significantly higher scores in both the I/R and HI/R groups compared to all other groups (*P* < 0.01), and decreased by all inhibitors (*P* < 0.01) (Figure 3A).

### Comparison of Brain Water Content and Infarct Size in Experimental Rats

Significant difference of brain water content existed among experimental groups [*F*(9,20) = 20.794, *P* < 0.000], and *post hoc* testing showed a significantly higher water content in the I/R group and HI/R group compared to the C group, especially in the latter (*P* < 0.01). Furthermore, the water content decreased significantly in the A, P, O, and D groups, as opposed to the HI/R group (*P* < 0.05 or 0.01) (Figure 3B). TTC staining showed cerebral infarctions in rats that received I/R operation. Statistical analyses showed significant difference of infarction size among experimental groups [*F*(6,14) = 358.431, *P* < 0.000], and *post hoc* testing showed the first and second largest infarction size of cerebrum tissue in the HI/R and I/R groups, respectively (*P* < 0.01), whereas the A, P, O, N, and D groups was obviously lower (*P* < 0.01) (Figure 3C).

### Changes in Mitochondrial Morphology of Brain Tissue

**FIGURE 4:**
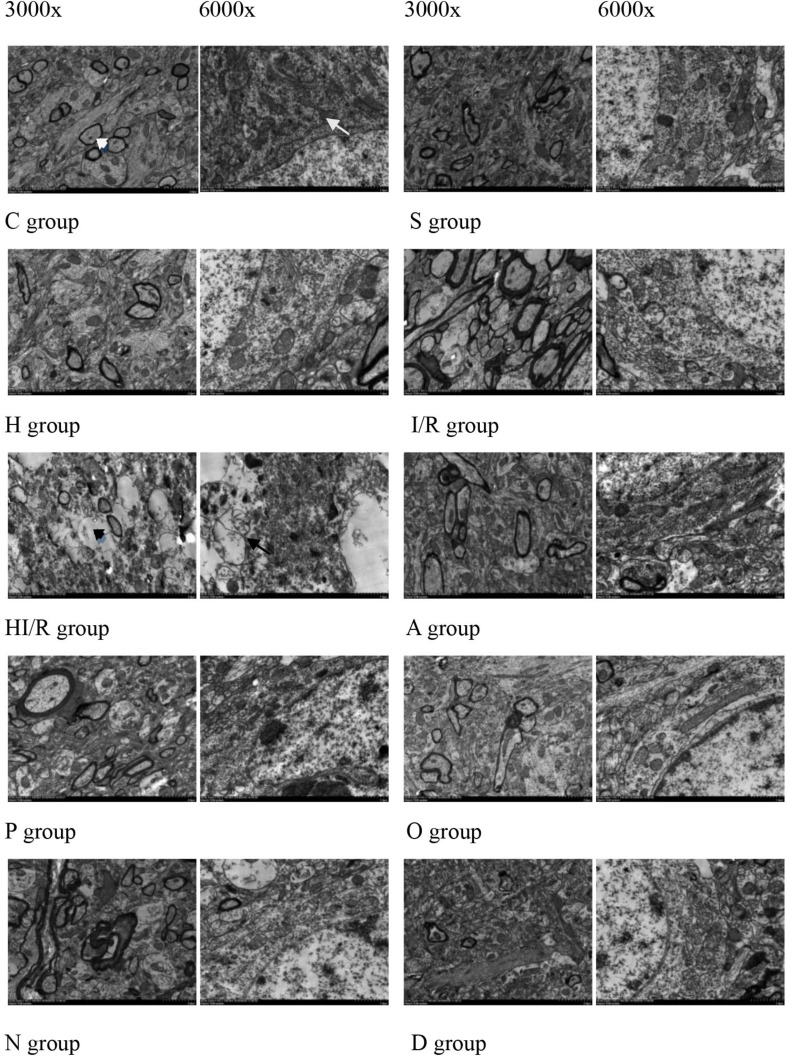
Morphology of cell, myelin sheath, nuclear membrane and mitochondria of brain tissue of the rats 24 h after I/R or not. Abundant round or oval mitochondrial (arrowheads) and clear crista (arrows), myelin sheath and nuclear membrane appeared in the rats of control (C) and sham operated groups (S). Partly impairment of myelin sheath and irregularity of nuclear membrane appeared in H group (H). Myelin sheath destroyed, nuclear membrane disappeared (black arrows), and organelle (black arrowheads) decreased in the rats received I/R, especially I/R combined with HHcy (HI/R). Evident impaired but lesser morphological change in inhibitor groups (A, P, O, N, and D).

Transmission electron microscope were used to observe changes in mitochondrial morphology. As a result, the C and S groups presented a clear myelin sheath and nuclear membrane, as well as abundant round or oval mitochondria with clear crista in the cytoplasm of the cerebral tissue. In contrast, a partly impaired myelin sheath and irregular nuclear membrane were observed in the H group, even though the mitochondria remained unchanged. The morphological change was most evident in the HI/R group, which demonstrated severe edema, vague crista, partial vacuolization, a destroyed myelin sheath, the disappearance of the nuclear membrane and a decrease in organelles. Similar changes, even though to a lesser extent, were also seen in the I/R group. Finally, fewer changes were observed in the A, P, O, N, and D groups (Figure 4).

### Change of Inflammatory Factors in Experimental Rats

**FIGURE 5:**
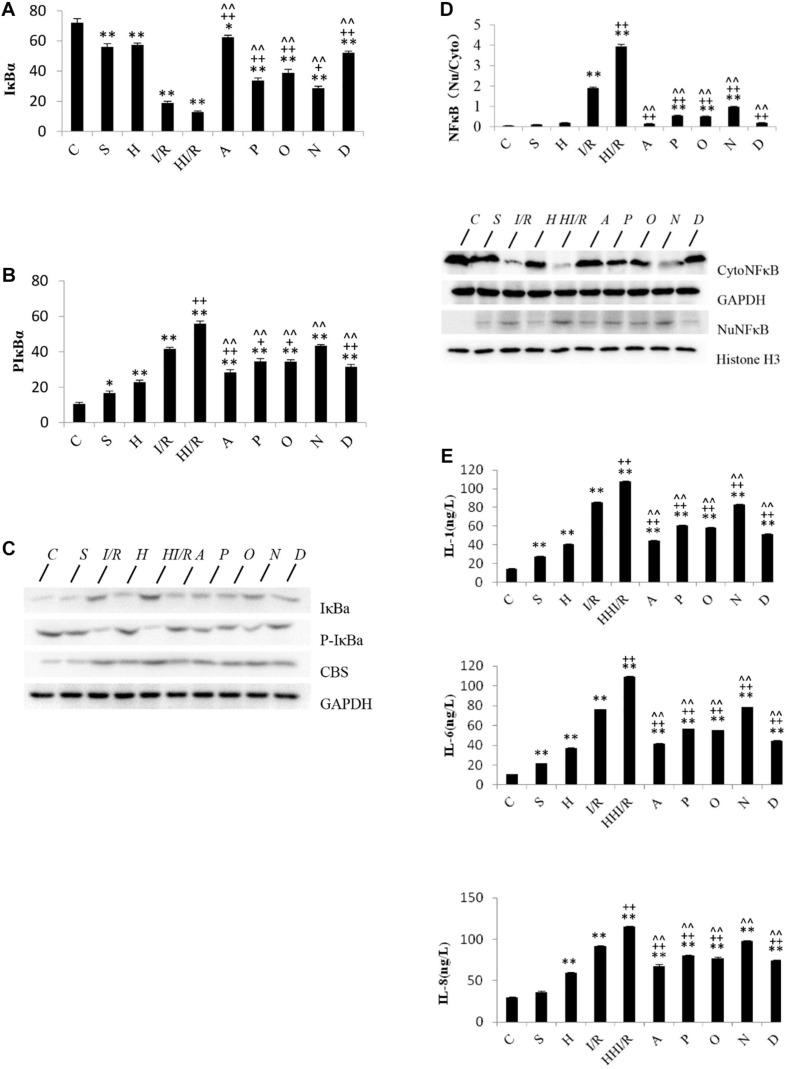
Changes of IκBα, p-IκBα, and NFκB translocation, IL-1, IL-6, and IL-8 levels in cerebrum tissue in rats 24 h after I/R or not. **(A)** IκBα levels decreased significantly in all other groups, compared to the C group (*P* < 0.05 or 0.01). The first and second lowest IκBα levels appeared in the HI/R and I/R groups, respectively (*P* < 0.01), and increased by all inhibitors (*n* = 3, *P* < 0.05 or 0.01). **(B)** The first and second lowest P-IκBα levels appeared in the HI/R and I/R groups, respectively (*P* < 0.01). Significant decrease of p-IκBα levels appeared in all inhibitor groups than in the HI/R group, but appeared in A, P, O, and D groups when compared to the I/R group (*n* = 3, *P* < 0.05 or 0.01). **(C)** The blot band demonstrated changes of IκBα, p-IκBα, and CBS protein levels in each group. GAPDH was used as normalized protein. **(D)** Translocation of NFκB levels into nuclear evidently increased in I/R and HI/R groups compared to control group, and decreased by all inhibitors (*n* = 3, *P* < 0.01). The blot band showed changes of Cyto NFκB and Nu NFκB protein levels in each group. GAPDH and Histone H3 were used as normalized protein, separately. **(E)** The IL-1, IL-6, and IL-8 levels in I/R and HI/R groups significantly increased compared to control groups, especially in the latter, and evidently decreased in A, P, O, and D groups than that in HI/R group and I/R group (*n* = 3, *P* < 0.01). ^∗∗^ or ^∗^ means *P* < 0.01 or 0.05 versus control, ^++^or ^+^ means *P* < 0.01 or 0.05 versus I/R group, and ^∧∧^ or ^∧^ means *P* < 0.01 or 0.05 versus HI/R group, analyzed by one-way ANOVA and Bonferroni *post hoc* test.

Statistical analyses showed significant difference of IκBα levels among experimental groups [*F*(9,20) = 134.199, *P* < 0.000], and *post hoc* testing demonstrated significantly decreased IκBα levels in all other groups, compared to the C group (*P* < 0.05 or 0.01). Specifically, the first and second lowest IκBα levels were observed in the HI/R and I/R groups, respectively, and increased by all inhibitors (*P* < 0.05 or 0.01) (Figure 5A). With regards to the p-IκBα levels, significant difference existed among experimental groups [*F*(9,20) = 113.955, *P* < 0.000], and *post hoc* testing showed the first and second highest levels in the HI/R and I/R groups, respectively (*P* < 0.01). Significant decrease appeared in all inhibitor groups compared to the HI/R group, but only appeared in A, P, O, and D groups as opposed to the I/R group (*P* < 0.05 or 0.01) (Figure 5B). The western blot bands of IκBα and p-IκBα are displayed in Figure 5C. There were significant difference of NF-κB translocation among experimental groups [*F*(9,20) = 1040.637, *P* < 0.000], and *post hoc* testing showed higher translocation in the I/R and HI/R groups (*P* < 0.01), and decreased by all inhibitors (*P* < 0.01) (Figure 5D). The blot bands of NF-κB are demonstrated in Figure 5D. Finally, significant difference of the IL-1, IL-6, and IL-8 levels existed among experimental groups [*F*(9,20) = 16900.86, 17695.045, and 336.312, respectively, *P* < 0.000], and *post hoc* testing showed significant increase in the IL-1, IL-6, and IL-8 levels in all groups compared to C group (*P* < 0.000). The highest level was observed in the HI/R group, which was closely followed by the I/R group (*P* < 0.000), and evidently decreased in A, P, O, and D groups (*P* < 0.01) (Figure 5E).

### Change of Hcy and H_2_S Pathway in Experimental Rats

**FIGURE 6:**
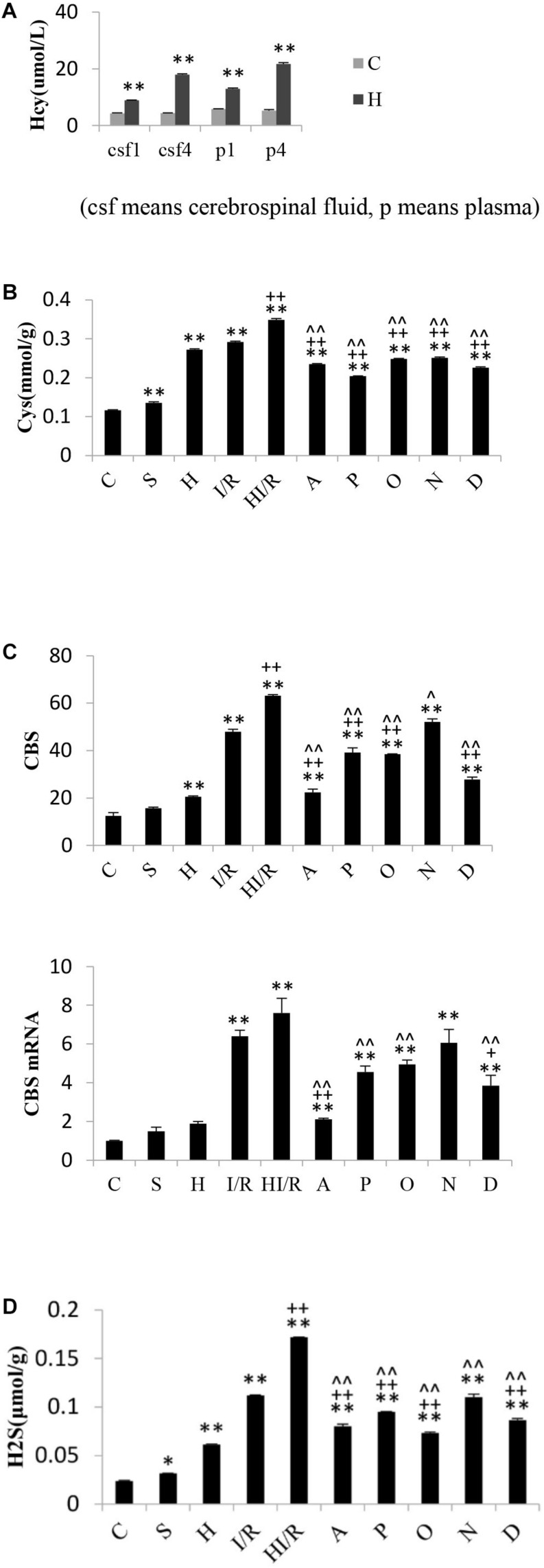
Changes of Hcy levels in rats fed for one and 4 weeks, and Cys, CBS, and H_2_S levels in rats 24 h after I/R or not. **(A)** Hcy levels in plasma and cerebrospinal fluid of rats received normal food or food added methionine for 1 week or 4 weeks. A higher Hcy level appeared in H group than that in C group (*n* = 5, *P* < 0.01). **(B)** Cys levels in I/R and HI/R groups significantly increased compared to C group, especially in the latter (*P* < 0.01), and evidently decreased in A, P, O, N, and D groups than that in HI/R group and I/R group (*n* = 3, *P* < 0.01). **(C)** CBS levels in I/R and HI/R groups significantly increased compared to control groups, especially in the latter (*P* < 0.01), and evidently decreased in A, P, O, and D groups than that in HI/R group and I/R group (*n* = 3, *P* < 0.01). CBS mRNA levels in I/R and HI/R groups significantly increased compared to control groups, especially in the latter (*P* < 0.01), and evidently decreased in A and D groups than that in HI/R group and I/R group (*n* = 3, *P* < 0.01). **(D)** H_2_S levels in I/R and HI/R groups significantly increased compared to control groups, especially in the latter, and evidently decreased in A, P, O, and D groups than that in HI/R group and I/R group (*n* = 3, *P* < 0.01). ^∗∗^ or ^∗^ means *P* < 0.01 or 0.05 versus control, ^++^or ^+^ means *P* < 0.01 or 0.05 versus I/R group, and ^∧∧^or ^∧^ means *P* < 0.01 or 0.05 versus HI/R group, analyzed by one-way ANOVA and Bonferroni *post hoc* test.

Significant difference of Hcy levels in plasma and cerebrospinal fluid existed among experimental groups [*F*(3,16) = 527.566 and 1563.3, respectively, *P* < 0.000], and *post hoc* testing showed significantly higher Hcy levels in the rats received food with methionine than those fed with normal food (*P* < 0.01) (Figure 6A). As far as Cys levels, significant difference existed among experimental groups [*F*(9,20) = 1017.961, *P* < 0.000], and *post hoc* testing showed the lowest concentration in the C group, whereas the HI/R, I/R and H groups had the top three highest levels, respectively (*P* < 0.000). Compared to HI/R group and I/R group, Cys levels evidently decreased in all inhibitor groups (*P* < 0.01) (Figure 6B). There were significant difference of CBS levels among experimental groups [*F*(9,20) = 183.349, *P* < 0.000], and *post hoc* testing showed CBS levels in I/R and HI/R groups significantly increased compared to the control group, especially in the latter (*P* < 0.01), and evidently decreased in all inhibitors than in HI/R group but only in A, P, O, and D groups as opposed to I/R group (*P* < 0.05 or 0.01) (Figure 6C). Significant difference of CBS mRNA levels existed among experimental groups [*F*(9,20) = 32.116, *P* < 0.000], and *post hoc* testing demonstrated higher CBS mRNA levels appeared in I/R and HI/R groups compared to the control group, especially in the latter (*P* < 0.01), and decreased in A, P, O, and D groups compared to HI/R group but only in A and D groups as opposed to I/R group (Figure 6C). The blot band of CBS is listed in Figure 5C. Similarly, significant difference of H_2_S levels existed among experimental groups [*F*(9,20) = 919.121, *P* < 0.000], and *post hoc* testing showed the first and second highest concentrations in the HI/R and I/R groups, and evidently decreased by all inhibitors than in HI/R group but only in A, P, O, and D groups compared to I/R group (*P* < 0.01) (Figure 6D).

### Change of Mitochondria Function, ROS and Ca^2+^ Levels in Experimental Rats

**FIGURE 7:**
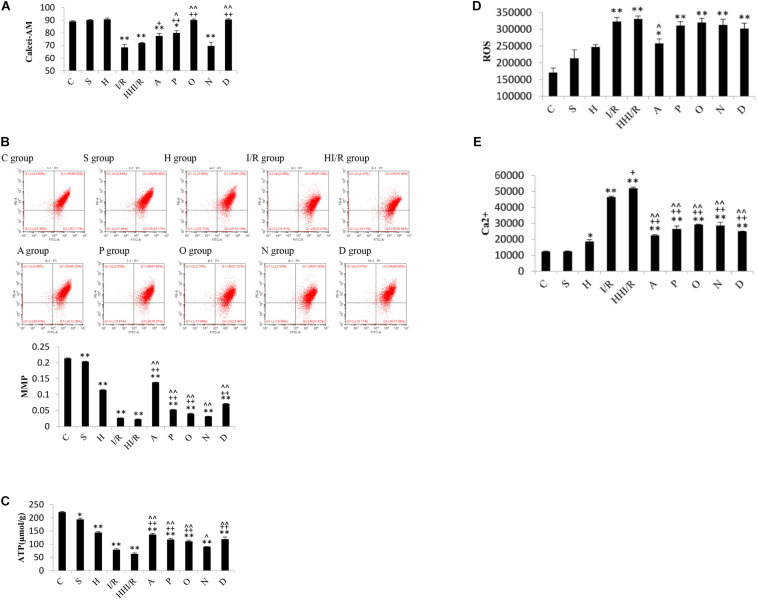
Changes of MPTP, MMP, ATP, ROS, and Ca^2+^ levels in rats 24 h after I/R or not. **(A)** Calcei-AM levels evidently decreased in I/R and HI/R groups compared to control group, and increased significantly by P, O, and D groups (*n* = 3, *P* < 0.05 or 0.01). **(B)** MMP levels in I/R and HI/R groups significantly decreased compared to control groups, especially in the latter, and evidently increased in A, P, O, and D groups than that in HI/R group and I/R group (*n* = 3, *P* < 0.01). **(C)** The ATP levels in I/R and HI/R groups significantly decreased compared to control groups, especially in the latter, and evidently increased in A, P, O, and D groups than that in HI/R group and I/R group (*n* = 3, *P* < 0.01). **(D)** ROS levels increased significantly in I/R or HI/R than that in control groups (*P* < 0.01). **(E)** Ca^2+^ levels in I/R and HI/R groups significantly increased compared to control groups, especially in the latter, and evidently decreased in A, P, O, N, and D groups than that in HI/R group and I/R group (*n* = 3, *P* < 0.01). ^∗∗^ or ^∗^ means *P* < 0.01 or 0.05 versus control, ^++^or ^+^ means *P* < 0.01 or 0.05 versus I/R group, and ^∧∧^or ^∧^ means *P* < 0.01 or 0.05 versus HI/R group, analyzed by one-way ANOVA and Bonferroni *post hoc* test.

Through flow cytometry, the decrease in Calcei-AM, the main component of the MPTP assay kit (i.e., a strong fluorescent agent), means the increase in MPTP can be assessed. As a result, significant difference of concentration of Calcei-AM existed among experimental groups [*F*(9,20) = 36.066, *P* < 0.000], and *post hoc* testing showed evidently lower concentration in the I/R and HI/R groups compared to the control group, and increased concentration in A, P, O, and D groups than in I/R group but only in P, O, and D groups compared to HI/R group (*P* < 0.05 or 0.01) (Figure 7A). As far as MMP levels, significant difference existed among experimental groups [*F*(9,20) = 4488.385, *P* < 0.000], and *post hoc* testing showed lower levels in I/R and HI/R groups compared to control groups, especially in the latter, and evidently increased in inhibitors groups than in HI/R group but in A, P, O, and D groups as opposed to I/R group (*P* < 0.01) (Figure 7B). Similar change of ATP levels existed among experimental groups [*F*(9,20) = 123.192, *P* < 0.000], and *post hoc* testing showed decreased levels in HI/R group and I/R group (*P* < 0.01) (Figure 7C). Significant difference of ROS levels existed among experimental groups [*F*(9,20) = 13.75, *P* < 0.000], and *post hoc* testing showed higher levels in the I/R and HI/R groups than in the C group (*P* < 0.01), and decreased in the A group compared to HI/R group (*P* = 0.05) (Figure 7D). With regards to the Ca^2+^ level, significant difference existed among experimental groups [*F*(9,20) = 164.582, *P* < 0.000], and *post hoc* testing found highest level in the HI/R group (*P* < 0.01), followed by the I/R group (*P* < 0.01), and evidently decreased in all inhibitor groups compared to HI/R group and I/R group (*P* < 0.01) (Figure 7E).

## Discussion

Multiple evidences suggested a definite relationship between elevated plasma Hcy levels and the risk of cardiocerebro-vascular diseases (McCully, 1969; Castro et al., 2006; Maron and Loscalzo, 2009; Ji et al., 2013; Zhao et al., 2017). In a previous study, we observed elevated Hcy levels to be associated with poor prognosis in acute ischemic cerebral infarction patients (Ji et al., 2015). Similarly, various experimental evidences indicated a pathogenic role for Hcy (Kim et al., 1987; Klancnik et al., 1992; Jellinger, 2013; Krishna et al., 2013; Currò et al., 2014; Pushpakumar et al., 2014). A close relationship between HHcy and multiple kinds of neurological diseases has been established. To further explore the effects of HHcy on acute ischemic brain injury and the underlying mechanisms, the present study was performed on OGD plus NaSH-treated SH-SY5Y cells and rat models of I/R and HHcy.

Hcy and Cys are substrates of the CBS and CSE and are thus involved in the endogenous production of H_2_S (Griffith, 1987). Although H_2_S is the third endogenously produced gasotransmitter and is considered as an important cell signaling molecule, its physiological levels in the body remain to be defined (Goodwin et al., 1989; Warenycia et al., 1989; Savage and Gould, 1990; Han et al., 2006; Ishigami et al., 2009; Levitt et al., 2011). Under the physiologic condition, catabolism of methionine causes an increased Hcy degradation to Cys via the transsulfuration pathway, which spontaneously decomposes and releases H_2_S, and then H_2_S is rapidly catabolized by the brain tissue (Furne et al., 2008). Therefore, the brain H_2_S level is generally very low at this time. However, the brain H_2_S level may increase evidently under pathological condition. In a previous study, Julie et al. observed the anaerobic incubation of brain tissue yielded to higher H_2_S release rates than the aerobic incubation at all L-cysteine concentrations (Furne et al., 2008). In the present study, H_2_S levels in the brain tissue greatly increased to 110 and 171 nM in the rats under I/R and I/R combined with HHcy, respectively, as opposed to that in control group (24 nM). In concordance, the Cys levels also increased in rats that received either the I/R treatments alone or a combination of I/R and HHcy. To conclude, obvious increases in the H_2_S concentration were identified in the brain tissue following an ischemic/reperfusion situation, especially when this was accompanied by an elevated HHcy level.

Several effects associated with H_2_S have been reported in the pathophysiological realm. The physiological concentration of H_2_S (i.e., low μM) was reported to play cytoprotective effects through the following processes: activation of sarcolemmal KATP channels, activation of the protein kinase C, anti-apoptotic effects and modulation of cellular respiration (Stipanuk et al., 2002). Lower levels of H_2_S were identified to stimulate mitochondrial function, whereas higher level were associated with the inhibition of the cytochrome c oxidase, causing the mitochondrial respiration as a state of suspended animation in a dose-dependent manner (Furne et al., 2001; Calvert et al., 2010). Furthermore, supratherapeutic concentrations of H_2_S were shown to irreversibly depress mitochondrial respiration, affecting ATP synthesis and further contributing to I/R injury. Previous study found H_2_S played both stimulatory and inhibitory effects during the autophagy process (Wang et al., 2017). Its toxic effects are usually observed at relatively high concentrations, i.e., non-physiological levels (high μM to mM), which limits its clinical usefulness for treating patients (Stipanuk et al., 2002). As the dose-response curve of H_2_S is bell-shaped, the effects of this compound switch from protective to deleterious based on both the environmental conditions and concentrations (Kumar and Sandhir, 2018). Specifically, while lower concentrations stimulate several physiological processes, including mitochondrial function and antioxidant effects, higher concentrations lead to cell death or other adverse effects.

In the *in vitro* phase of the present study, high concentrations of the NaSH treatment (1,500 μM) were found to cause an increase in the levels of intracellular ROS and MPTP, release of cytochrome C, as well as a decrease in the MMP of SH-SY5Y cells under OGD. In contrast, low concentrations of NaSH treatment (100 μM) appeared beneficial. These reveal high levels of H_2_S exacerbate injury to cells under OGD although low levels demonstrate protective effects. *In vivo* phase of the study, the concentrations of H_2_S greatly increased in the acute ischemia or when it was combined with HHcy, and the overproduction of H_2_S produced a negative impact (Panagaki et al., 2019), such as a worsening in the brain tissue. Specifically, the infarction sizes of the cerebral tissues enlarged, while their water content increased. Furthermore, the energy metabolism of cells was impaired and ATP production decreased. This was followed by Ca^2+^ influx and ROS accumulation. The inflammatory reaction appeared and mitochondrial morphology and function were destroyed. Finally, cytochrome c was released from the mitochondria, which in turn induced cell apoptosis. Such pathological changes were especially obvious in rats that received the combination of I/R and HHcy, suggesting that acute ischemic brain tissue injury deteriorated when it encountered a high Hcy concentration. Higher levels of H_2_S probably played a critical role during the pathological process supported by both *in vitro* and *in vivo* works of the study.

To further explore the possible mechanism underlying the involvement of H_2_S and HHcy in the acute ischemic injury, we added several inhibitors of CBS, CSE, Hcy, H_2_S, and NF-κB to the experimental groups. Specifically, both AOAA and HA were used as antagonists of CBS, whereas PAG was employed as an inhibitor of CSE. In contrast, the *N*-anandamide and PDTC antagonists were aimed for Hcy and NF-κB, respectively. Our results suggested that all inhibitors lessened the neurological function impairment, inflammatory reaction, mitochondria damage and decreased energy metabolism caused by acute ischemia and HHcy. While the AOAA, HA and PAG inhibitors showed obvious effects on the amelioration of the increased CBS, Cys, and H_2_S levels in the HHI/R rats, PDTC was more effective on alleviating the release of inflammatory factors. Interestingly, inhibiting CBS, CSE, and Hcy was accompanied by a decrease in inflammatory factors, whereas inhibiting NF-κB was associated with a reduction in the CBS, Cys, and H_2_S levels. Therefore, we here propose both the coexistence and interaction of H_2_S overproduction and inflammatory factors to be one of the pathological mechanisms underlying the HHI/R process. Furthermore, the AOAA inhibitor was suggested to be extremely useful in modifying both the H_2_S overproduction and the inflammatory reaction (Bento et al., 2016). In contrast, the *N*-anandamide inhibitor was less valuable, as it lessened the pathological changes in HHI/R rats only. In addition, mitochondrial function impairment was also involved in the pathological mechanism under HHI/R. In fact, inhibiting NF-κB resulted in a reduction of mitochondrial function impairment in HHI/R rats, whereas blocking either CBS or CSE only partly rectified the impairment. Therefore, it is reasonable to consider that H_2_S may indirectly impair mitochondrial function by increasing the inflammatory reaction in the HHI/R condition.

## Conclusion

To summarize, H_2_S overproduction and inflammatory factors are involved in the pathological changes occurring after acute ischemic brain injury and HHcy. Furthermore, mitochondrial dysfunction is also involved in the pathological process. Future studies in this direction are warranted to provide instructive guidance for targeted medicine development.

## Data Availability Statement

The original contributions generated for this study are included in the article/supplementary material, further inquiries can be directed to the corresponding authors.

## Ethics Statement

The animal study was reviewed and approved by the Institutional Animal Care and Use Committee of the University of Zhengzhou, School of Medicine.

## Author Contributions

YJ: conceptualization, methodology, writing – review and editing, project administration, and funding acquisition. YL: supervision, resources, and validation. ZZ: investigation and writing – original draft. PL: formal analysis and investigation. YX: data curation, visualization, and investigation. All authors contributed to the article and approved the submitted version.

## Conflict of Interest

The authors declare that the research was conducted in the absence of any commercial or financial relationships that could be construed as a potential conflict of interest.
